# UVA Photoactivation of Harmol Enhances Its Antifungal Activity against the Phytopathogens *Penicillium digitatum* and *Botrytis cinerea*

**DOI:** 10.3389/fmicb.2017.00347

**Published:** 2017-03-07

**Authors:** Gabriela M. Olmedo, Luciana Cerioni, María M. González, Franco M. Cabrerizo, Sabrina I. Volentini, Viviana A. Rapisarda

**Affiliations:** ^1^INSIBIO (CONICET, UNT), Instituto de Química Biológica “Dr. Bernabé Bloj”, Facultad de Bioquímica, Química y Farmacia, UNTTucumán, Argentina; ^2^IIB-INTECH – UNSAM-CONICETBuenos Aires, Argentina

**Keywords:** β-carboline, alkaloids, photosensitization, reactive oxygen species, cellular damage

## Abstract

Phytopathogenic fungi responsible for post-harvest diseases on fruit and vegetables cause important economic losses. We have previously reported that harmol (1-methyl-9H-pyrido[3,4-b]indol-7-ol) is active against the causal agents of green and gray molds *Penicillium digitatum* and *Botrytis cinerea*, respectively. Here, antifungal activity of harmol was characterized in terms of pH dependency and conidial targets; also photodynamic effects of UVA irradiation on the antimicrobial action were evaluated. Harmol was able to inhibit the growth of both post-harvest fungal disease agents only in acidic conditions (pH 5), when it was found in its protonated form. Conidia treated with harmol exhibited membrane integrity loss, cell wall disruption, and cytoplasm disorganization. All these deleterious effects were more evident for *B. cinerea* in comparison to *P. digitatum*. When conidial suspensions were irradiated with UVA in the presence of harmol, antimicrobial activity against both pathogens was enhanced, compared to non-irradiated conditions. *B. cinerea* exhibited a high intracellular production of reactive oxygen species (ROS) when was incubated with harmol in irradiated and non-irradiated treatments. *P. digitatum* showed a significant increase in ROS accumulation only when treated with photoexcited harmol. The present work contributes to unravel the antifungal activity of harmol and its photoexcited counterpart against phytopathogenic conidia, focusing on ROS accumulation which could account for damage on different cellular targets.

## Introduction

Several pre- and post-harvest diseases are caused by the attack of the phytopathogenic fungus *Botrytis cinerea* in more than 200 plant species (Elad and Evensen, 1995). Other fungus that has adverse effects on crop yields and quality is *Penicillium digitatum*, the causal agent of green mold, the most common post-harvest disease of citrus fruit (Holmes and Eckert, 1999). In agricultural practices, fungicides such as imazalil, thiabendazole and dicarboximide, are extensively used to control diseases provoked by these fungi. The continuous use of commercial fungicides has resulted in environmental contamination and the appearance of resistant strains of both phytopathogens (Latorre et al., 1994; Palou et al., 2002). The discovery of antimicrobial compounds to control fungal diseases of economic importance in agriculture remains a major scientific challenge. In this regard, natural products isolated from plants are continuously being evaluated for their antimicrobial activity and result in promising alternatives to commercial fungicides (Grayer and Kokubun, 2001; Soylu et al., 2010; Gatto et al., 2011; Sayago et al., 2012).

β-carbolines (βCs) comprise a class of alkaloids that are widely distributed in nature, including plants, foodstuffs, marine creatures, insects, mammalians, human tissues and body fluids. βCs are a large group of heterocyclic compounds with a 9H-pyrido[3,4-b]indole structural unit (Gonzalez et al., 2009a) that were originally isolated from *Peganum harmala* (Zygophyllaceae, Syrian Rue; Cao et al., 2007). These compounds are of great interest due to their antitumor, antiviral, antimicrobial and antiparasitic activities (Kobayashi et al., 1994; Di Giorgio et al., 2004; Cao et al., 2007; Alomar et al., 2013). They are also recognized as photochemically active substances. Upon UVA photoexcitation, these alkaloids are able to photoinduce damage on biologically relevant macromolecules (Hazen and Gutierrez-Gonzalvez, 1988; Gonzalez et al., 2010, 2012a,b; Vignoni et al., 2013, 2014) as well as to inactivate bacteria and viruses (McKenna and Towers, 1981; Hudson et al., 1986).

It has been previously reported that, among six βCs tested, harmol exhibited the highest inhibitory effect on *P. digitatum* and *B. cinerea* (Olmedo et al., 2017). At a concentration of 1 mM, this full aromatic βC provoked a complete germination inhibition of *B. cinerea* and *P. digitatum* conidia. For both pathogens, membrane permeabilization and significant reduction in conidia infectivity were detected. Thus, to characterize the antifungal activity of harmol against *P. digitatum* and *B. cinerea*, we investigated deleterious effects on several cellular features related to viability of conidia. The antifungal activity of harmol after UVA irradiation (i.e., photodynamic effect) was evaluated.

## Materials and Methods

### Chemicals, p*K*_a_ Determination and Preparation of Stock Solutions

Harmol from Sigma-Aldrich Co. (>98%) was used without further purification. p*K*_a_ values at room temperature were determined from changes in UV-vis absorption spectra of aqueous solutions of harmol, following the procedure described elsewhere (Cabrerizo et al., 2004). For antifungal assays, harmol stock solution was prepared in dimethyl sulfoxide (DMSO, Sigma-Aldrich Co.) and its concentration was calculated using the 𝜀_320nm_ = 18965 M^-1^ cm^-1^, as previously described by Olmedo et al. (2017).

### Fungal Isolates, Growth Conditions, and Preparation of Conidial Suspension

Fungal isolates were obtained from naturally infected fruit in Tucumán (Argentina). *P. digitatum* was isolated from lemons (Cerioni et al., 2009) and *B. cinerea* from blueberries (Olmedo et al., 2017). Both strains have been previously deposited with codes ICFC 842/15 and ICFC 841/15 in the IIB-INTECH collection of Fungal Cultures (ICFC, from the Laboratory of Mycology and Mushroom Cultivation, IIB-INTECH, Chascomús, Argentina; WDCM data base reference: 826).

Fungal isolates were grown on potato dextrose agar (PDA) at 22 ± 1°C, in the dark for 10 or 7 days, in the case of *P. digitatum* or *B. cinerea*, respectively. *B. cinerea* sporulation was induced by placing a sterile wood stick onto the growing colony and incubating for further 7 days (Olmedo et al., 2017).

Preparation of conidial suspensions (10^6^ conidia mL^-1^) was performed as previously described (Cerioni et al., 2009). Dilution media was sterile distilled water at pH 5, except for studies on effect of pH.

### Antifungal Activity of Harmol at Different pH

Conidial suspensions were prepared in sodium acetate-acetic acid buffer (pH 5) or Tris HCl buffer (pH 9). These suspensions were incubated in the presence of 1 mM harmol during 24 h. Controls containing buffer and DMSO without harmol were included. After incubation, harmol was removed by centrifugation at 10000 rpm for 10 min and replaced with the same volume of sterile distilled water. Five microliters of each suspension were spotted on PDA plates and colony formation was detected after 48 h of incubation. In addition, viability of conidia during treatments was evaluated, spreading serially diluted suspensions on PDA medium. Cell survival was quantified as colony forming units (CFU) mL^-1^ after 4 days of incubation at 22 ± 1°C.

### Ultrastructural Analysis of Conidia

For ultrastructural characterization by transmission electron microscopy, conidia were incubated for 24 h with DMSO (control) or 1 mM harmol. Afterward, samples were prepared as described by Cerioni et al. (2010). Observations were made with a Zeiss EM 109 transmission electron microscope from CIME (Centro Integral de Microscopía Electrónica, CONICET-UNT).

### Conidia Cell Wall Integrity Assay

Conidial suspensions treated with 1 mM harmol during 24 h were centrifuged and washed with sterile distilled water. Cell wall integrity was studied using the fluorescent dye Calcofluor White (CFW; Pringle, 1991), following a protocol adapted by Cerioni et al. (2010). Controls were performed in parallel, treating conidial suspensions with DMSO.

### Photodynamic Activity of Harmol

The enhancing effect of UVA irradiation on antifungal activity of harmol was investigated as follows. *P. digitatum* and *B. cinerea* conidial suspensions were placed in a 96-well polystyrene microtiter plate containing harmol at several concentrations. The plate was irradiated during 30 min at 22°C with a Philips HPW 8 W lamp emitting at 365 nm (bandwidth 20 nm). The dose rate at the irradiation site was 8 W/m^2^ (Spectrosense 2+ UV radiometer, Skye Instruments Ltd). A microplate containing identical treatments was placed at the same temperature, in the dark. After irradiation, both microplates were incubated during 24 h at 22°C. CFU mL^-1^ were counted and the percentage of viability was determined (Muniz-Paredes et al., 2016).

### Determination of Reactive Oxygen Species (ROS) Production

Conidial suspensions treated with harmol were irradiated or non-irradiated, as explained above. Control suspensions were treated with DMSO. After 24 h of incubation, suspensions were washed and resuspended in sterile distilled water. Reactive oxygen species (ROS) were determined with the H_2_DCFDA probe (Davidson et al., 1996; Halliwell and Whiteman, 2004), following the protocol adapted by Cerioni et al. (2010).

H_2_O_2_ *in situ* detection in conidia was performed using the 3,3′-diaminobenzidine (DAB) uptake method (Thordal-Christensen et al., 1997) with some modifications. Suspensions were exposed to 0.5 mg mL^-1^ DAB solution and incubated during 8 h in the dark. Conidia were observed with a microscope Olympus IX51 equipped with an Olympus digital camera. A reddish-brown reaction product indicates H_2_O_2_ presence.

### Statistical Analysis

All assays were performed three times, including three replicates for each condition. Data were subjected to analysis of variance followed by Tukey’s test (Infostat, 2013 version, for Windows). Differences of *p* value ≤ 0.05 were considered significant.

## Results

### pH-Dependent Antifungal Activity of Harmol

**FIGURE 1:**
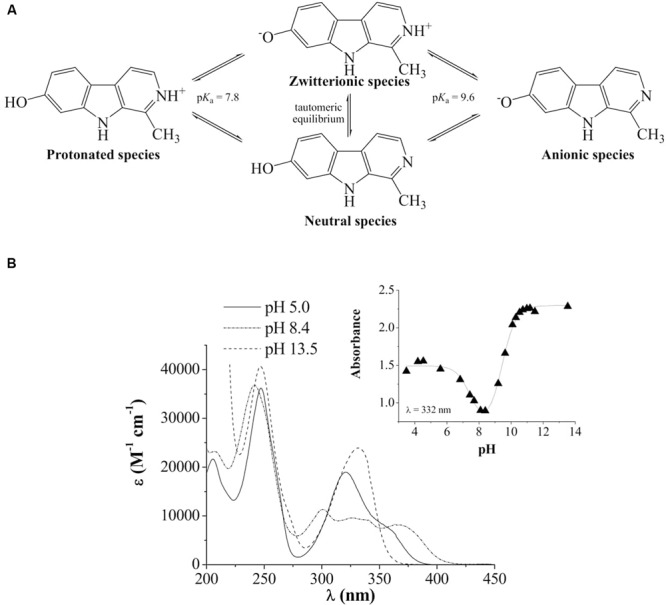
**(A)** Chemical structure and acid-base equilibria of harmol. **(B)** UV-visible spectra of harmol in acidic (pH 5), low-alkaline (pH 8.4) and alkaline (pH 13.5) aqueous solutions. *Inset:* representative example of the spectrophotometric titration curve recorded at the absorption wavelength of 332 nm.

**FIGURE 2:**
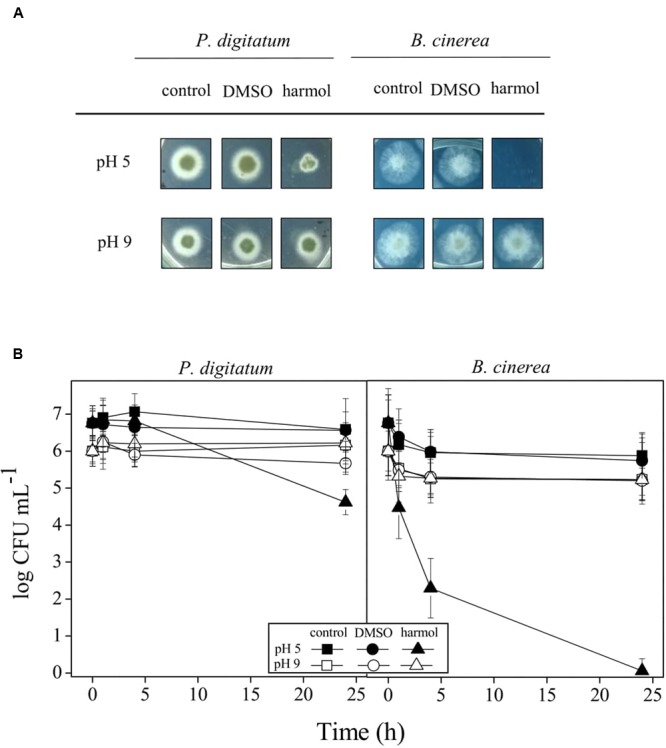
**Effect of pH on antifungal activity of harmol.** *P. digitatum* and *B. cinerea* conidia were treated for 24 h with 1 mM harmol at the indicated pH. Controls with or without DMSO were included. **(A)** After treatments, colony formation was assessed using PDA plates incubated for 48 h. Photographs are representative of three independent experiments. **(B)** Viability of conidia was determined by counting CFU mL^-1^ at the indicated times during the treatments with harmol. Three independent experiments were performed.

In aqueous solutions, in the pH range 2–13, full aromatic βCs show different acid–base equilibria (Gonzalez et al., 2009b). In the case of harmol, the most relevant equilibrium present under physiological pH involves the deprotonation of the pyridinic nitrogen (N-2), with an overall p*K*_a_ value of 7.8 (**Figure 1**). This p*K*_a_ value is in agreement, within the experimental error, with the value previously reported (Tomas et al., 1985). The other functional groups, i.e., the hydroxy-substituent placed at position 7 and the nitrogen of the indole ring, have p*K*_a_ values higher than 9.6. In order to ascertain the contribution of each physiologically relevant species (i.e., protonated and neutral) on the antifungal activity, the effect of 1 mM harmol against *P. digitatum* and *B. cinerea* conidia was evaluated in acidic (pH 5, where harmol is present at more than 99% in its protonated form) and alkaline (pH 9, where a mixture of neutral, zwitterionic and anionic species of harmol is present) conditions (**Figures 2A,B**). After 24 h of incubations with harmol at pH 9, CFU mL^-1^ remained unchanged for both pathogens. In contrast, at pH 5, harmol protonated species exhibited a significant antimicrobial effect. *P. digitatum* CFU mL^-1^ counts were twofold reduced compared to controls, while *B. cinerea* exhibited a complete loss of viability.

### Modifications in Ultrastructure of Conidia by Harmol

**FIGURE 3:**
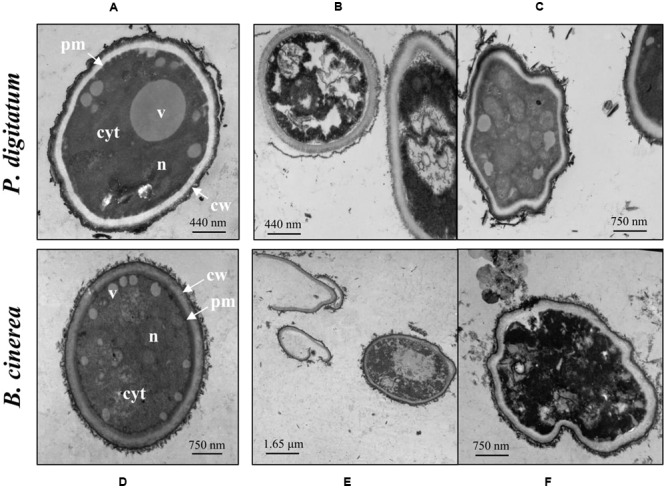
**Effect of harmol on ultrastructure of conidia.** Transmission electron micrographs of the indicated pathogens: **(A,D)** conidia in control treatment; **(B,C,E,F)** conidia treated with 1 mM harmol. Photographs are representative of three independent experiments. cyt, cytoplasm; v, vacuole; n, nucleus; pm, plasmatic membrane; cw, cell wall.

**Figure 3** shows TEM photomicrographs of *P. digitatum* and *B. cinerea* conidia treated with 1 mM harmol during 24 h. Almost all treated conidia exhibited unclear nuclei structures and disordered cytoplasms, revealing a severe cellular damage (**Figures 3B,E,F**). In some cases, significant distortion of the cell shape and/or loss of intracellular content were observed (**Figures 3C,F**). Samples of *B. cinerea* conidia treated with harmol exhibited cellular debris, indicating lysis (**Figures 3E,F**). In contrast, for both phytopathogens, conidia in control treatments showed a well-organized cytoplasm, nuclei, and visible vacuoles surrounded by well-defined envelopes (**Figures 3A,D**).

**FIGURE 4:**
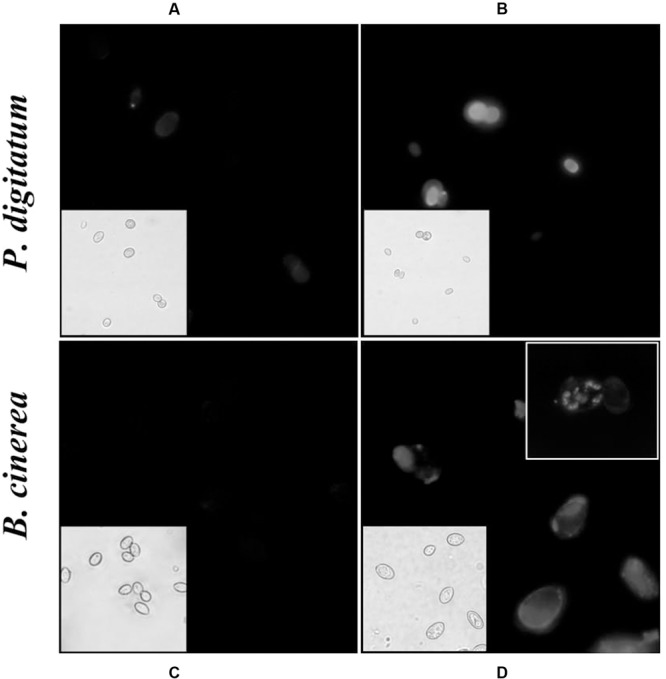
**Effect of harmol on integrity of the cell wall.** Conidia were exposed to 1 mM harmol during 24 h and incubated with CFW. Fluorescence microscopy images (100×) of conidia in control treatment **(A,C)**, and treated conidia **(B,D)**. The corresponding light microscopy images are shown for each panel. Photographs are representative of three independent experiments.

**Figure 4** shows fluorescence and light microscopy images of CFW stained cells. Conidia treated with harmol exhibited a stronger CFW fluorescence pattern on cell surface (**Figures 4B,D**) compared with the untreated controls (**Figures 4A,C**). In addition, bright spots were frequent throughout *B. cinerea* conidia treated with harmol (**Figure 4D**, inset).

### Enhancement of Antifungal Activity of Harmol by UVA Photoactivation

Some βC alkaloids have been described as efficient photosensitizers in acidic conditions (Gonzalez et al., 2009b, 2012a). Thus, toxicity of electronically photoexcited harmol was screened at pH 5, exposing the treatments to UVA irradiation (**Table 1**). The decrease in viability achieved in 30 min-irradiated treatments was different between both fungi, being *B. cinerea* the most sensitive. For this phytopathogen, photosensitized treatment required half of the harmol concentration to reach the same inhibitory effect compared to the non-irradiated counterpart treatment. Moreover, UVA irradiation in the presence of 0.1, 0.2 and 0.3 mM harmol, provoked a significant decrease in conidia survival. In respect to *P. digitatum*, dark and irradiated treatments resulted on viability of 1.15 and 0.5%, respectively. It is worth to note that conidia viability of both pathogens was not altered by a 30 min irradiation with UVA in the absence of harmol (data not shown).

**Table 1:** Photodynamic effect of harmol on viability of conidia.

	Viability (%)			
	*P. digitatum*		*B. cinerea*	
[mM]	harmol	harmol + UVA	harmol	harmol + UVA
–	100.00 ^a^	100.00 ^a^	100.00 ^a^	100.00 ^a^
0.1	97.40 ^a^	100.00 ^a^	63.80 ^b^	6.80 ^b#^
0.2	95.50 ^a^	83.20 ^c^	7.70 ^c^	1.50 ^c#^
0.3	84.20 ^a^	88.80 ^b^	1.20 ^d^	0.20 ^d#^
0.5	8.30 ^b^	1.00 ^d#^	0.02 ^d^	0.00 ^d^
1.0	1.50 ^b^	0.50 ^d#^	0.00 ^d^	0.00 ^d^

*Letters indicate significant differences (*p* ≤ 0.05) within columns. ^#^ indicates significant differences (*p* ≤ 0.05) between irradiated and non-irradiated treatments with the same harmol concentration.*

### ROS Accumulation after Incubation with Harmol

**FIGURE 5:**
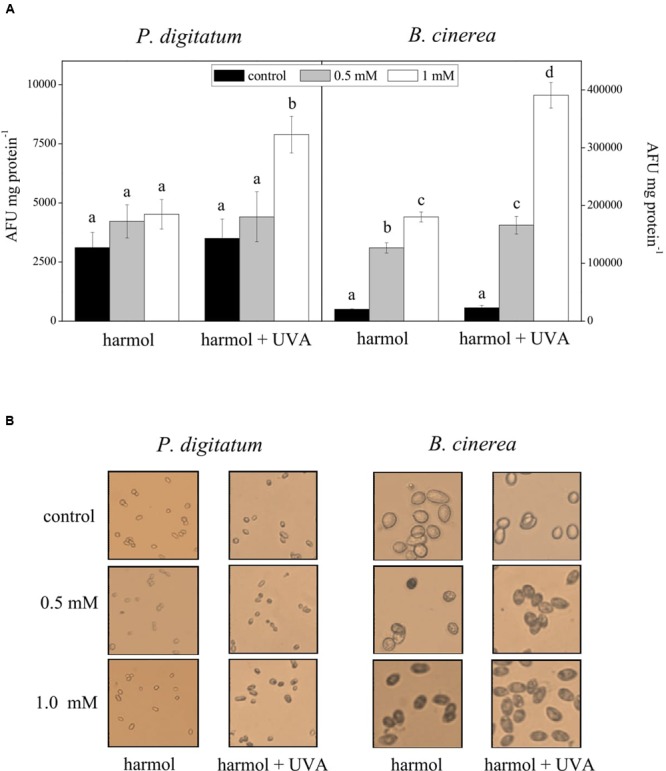
**Effect of harmol on production of intracellular ROS.** Conidia were treated with harmol at the indicated concentrations, and irradiated or non-irradiated with UVA. **(A)** ROS quantification using the H_2_DCFDA fluorescent probe. Data are the average ± SD of three experiments. For each pathogen, different letters indicate significant differences among treatments. AFU, arbitrary fluorescence units. **(B)** H_2_O_2_ *in situ* detection by staining with 3,3′-diaminobenzidine (DAB). Light microscopy photographs (40×) are representative of three independent experiments. H_2_O_2_ accumulation is visualized as dark conidia.

Intracellular ROS production was studied in conidia incubated with harmol at pH 5, irradiated with UVA or non-irradiated (**Figure 5**). For *P. digitatum*, the intracellular ROS content in non-irradiated treatments was similar to that of the controls (**Figure 5A**). In photoactivated treatments, a significant ROS accumulation was observed in conidia exposed to the highest harmol concentration (1 mM). In respect to *B. cinerea*, the exposure to harmol in the dark led to six- to eight-fold increments in the ROS production compared to controls without harmol. Furthermore, ROS production in *B. cinerea* conidia treated with photoactivated harmol was over 15 times higher than that of controls.

A significant DAB staining was observed in *B. cinerea* conidia treated with harmol, which indicates H_2_O_2_ accumulation (**Figure 5B**). Pigmentation was more evident when suspensions were exposed to UVA. In contrast, a lack of pigmentation in *P. digitatum* conidia was observed in all treatments, except in the conidial suspension treated with 1 mM harmol and exposed to UVA.

## Discussion

Harmol has antimicrobial properties against *P. digitatum* and *B. cinerea* (Olmedo et al., 2017). The exposure to this compound inhibited germination, mycelial growth, sporulation and residual infectivity of these microorganisms. In the present work, we characterized antifungal activity of harmol in terms of pH and conidial targets. An enhancement of harmol toxicity was achieved by photodynamic effect.

Our results indicate that antifungal activity of harmol strongly depends on pH. Protonated harmol was active against both phytopathogens, while the neutral, zwitterionic and/or anionic species lacked inhibitory ability. We consider that the interaction between harmol in its cationic form and the negatively charged microbial surfaces is probable to occur. In agreement, cationic species guides antimicrobial properties of different molecules (Boman, 1994; Ladokhin et al., 1997; Matsuzaki et al., 1997; Muñoz et al., 2006). In addition, it has been reported that changes in βCs electronic ground state distribution at different pH have a strong effect on chemical and binding properties of these compounds (Gonzalez et al., 2010, 2012a,b, 2014; Vignoni et al., 2013).

We have previously demonstrated that harmol at pH 5 induces membrane permeabilization in *P. digitatum* and *B. cinerea* cells (Olmedo et al., 2017). In CFW staining assays, performed at the same pH, bright spots and fluorescence increment were observed in harmol treated conidia. This suggests that cell wall integrity was altered, leading to the exposure of chitin, the main structural polysaccharide of internal wall. Namely, the fluorescence increment might be explained by the modification or loss in the outermost cell surface proteins, which expose internal cell wall components (Kubitschek-Barreira et al., 2013; Huang et al., 2016). Moreover, when conidia treated with harmol were observed by TEM, the most evident effect was wall distortion, with the consequent leak of cytoplasmic content at least in *B. cinerea*. It has been previously reported that dissolution or perturbation of wall structural polymers have adverse effects upon growth and differentiation of fungi (Poulose, 1992; Lorito et al., 1994; Soylu et al., 2007, 2010).

Previous reports stated that, upon UV light irradiation, βCs exhibit phototoxic properties (McKenna and Towers, 1981; Mori et al., 1998; Cao et al., 2007). Antifungal activity of harmol was severely improved when treatments were irradiated with UVA. In our irradiated approach, the complete inhibition of *B. cinerea* was achieved with lower concentrations compared to non-irradiated treatments. The eradication of this pathogen is important because *B. cinerea* causes disease at very low inoculum levels. For instance, it has been reported that tomato stems can be infected with only 10 conidia applied to the surface (O’Neill et al., 1997). On the other hand, 10^3^ *P. digitatum* conidia mL^-1^ remained viable after irradiated treatments. Nevertheless, this inoculum might be unable to infect fruit, considering data in our previous work (Olmedo et al., 2017). Our study provides useful knowledge for the design of novel antifungal compounds based on βCs skeleton, with a distinctive photodynamic behavior.

High ROS values in *B. cinerea* treated with harmol in non-irradiated condition agrees with the detected fungicidal action. In respect to *P. digitatum*, ROS accumulation did not occur, which is in accordance with the fungistatic effect previously reported (Olmedo et al., 2017). When conidia were irradiated in the presence of harmol, ROS production was enhanced for both phytopathogens with the consequent loss of viability. The ability of some βCs to generate ROS under UVA irradiation has been previously reported (Gonzalez et al., 2009b). It was proposed that H_2_O_2_ is formed by electron transfer to O_2_ yielding the superoxide anion (O_2_^●-^) and its spontaneous disproportionation to H_2_O_2_. Mechanistically, photodynamic effects can be a consequence of a direct reaction of the excited state of the photosensitizer with biomolecules and/or structural constituents of the fungi (i.e., Type-I reactions) or indirectly via ROS (i.e., Type-II reactions; Foote, 1991). Previous findings for other βC derivatives suggest that Type I reactions seem to be responsible for the damage generation (Gonzalez et al., 2010, 2012a). In the case of harmol, a similar pattern would be expected as the most probable mechanism of action. However, in view of our results, the Type-II reactions should not be discarded.

Taken together, our data suggest that the antifungal action of harmol may begin with electrostatic interactions between the protonated βC and conidial surface, which leads to the alteration of envelopes integrity, increment in ROS production, morphological damage, and cell collapse. UVA photoactivation improves the fungicidal action of harmol, allowing a decrease in the effective doses. The mode of action and the multiple cellular targets that may be affected by the drug prevent the appearance of resistant fungal strains. The combination of chemo- and photodynamic therapies represents an effective treatment to inactivate microorganisms. The use of harmol in the design of antifungals absorbing in the far UVA could be a good alternative for both, pre-harvest treatments (considering the sun as an UVA source) and post-harvest treatments (including UVA lamps in packing houses).

## Author Contributions

GO, LC, MG, FC, SV, and VR designed the experiments. GO, LC, and MG performed the experiments. GO, LC, MG, FC, SV, and VR analyzed the data. GO, FC, SV, and VR drafted the manuscript. All authors read and approved the final manuscript.

## Conflict of Interest Statement

The authors declare that the research was conducted in the absence of any commercial or financial relationships that could be construed as a potential conflict of interest.

The reviewer RT and handling Editor declared their shared affiliation and the handling Editor states that the process nevertheless met the standards of a fair and objective review.
